# Infection Risk, Mortality, and Hypogammaglobulinemia Prevalence and Associated Factors in Adults Treated with Rituximab: A Tertiary Care Center Experience

**DOI:** 10.3390/clinpract13060115

**Published:** 2023-10-25

**Authors:** Moustafa S. Alhamadh, Thamer S. Alhowaish, Alaa Mathkour, Bayan Altamimi, Shahd Alheijani, Abdulrahman Alrashid

**Affiliations:** 1 College of Medicine, King Saud bin Abdulaziz University for Health Sciences, Ministry of the National Guard-Health Affairs, Riyadh 14611, Saudi Arabia; 2 King Abdullah International Medical Research Center, Ministry of the National Guard-Health Affairs, Riyadh 11481, Saudi Arabia; alhowaish092@ksau-hs.edu.sa (T.S.A.);; 3 Department of Neurology, King Abdulaziz Medical City, Ministry of the National Guard-Health Affairs, Riyadh 11426, Saudi Arabia; 4 Ministry of Health, Riyadh 12613, Saudi Arabia; 5 Department of Medicine, Division of Rheumatology, King Abdulaziz Medical City, Ministry of the National Guard-Health Affairs, Riyadh 11426, Saudi Arabia; 6 College of Medicine, Alfaisal University, Riyadh 11533, Saudi Arabia

**Keywords:** rituximab, hypogammaglobulinemia, immunoglobulin, CD20, immunosuppression, autoimmunity, hematological malignancies

## Abstract

Background: Rituximab is a human monoclonal antibody directed against the B-cell transmembrane protein CD20. Although well-tolerated, given its mechanism of action, rituximab can induce a state of severe immunosuppression, increasing the risk of opportunistic and fulminant infection and mortality. Aim: To evaluate the risk of infection, mortality, and hypogammaglobulinemia and their associated factors among rituximab receivers. Method: This was a single-center retrospective cohort study of adults treated with rituximab for various indications. Hypogammaglobulinemia was defined by a cut-off value below the normal limit (an IgG level of <7.51 g/L, an IgM level of <0.46 g/L, and/or an IgA level of <0.82 g/L). Patients who met the definition of hypogammaglobinemia solely based on IgA were excluded. Severe infection was defined as any infection that required intensive care unit admission. Results: A total of 137 adults with a mean age of 47.69 ± 18.86 years and an average BMI of 28.57 ± 6.55 kg/m^2^ were included. Hematological malignancies and connective tissue diseases were the most common primary diagnoses for which rituximab was used. More than half of the patients received the 375 mg/m^2^ dose. Rituximab’s mean cumulative dose was 3216 ± 2282 mg, and the overall mortality rate was 22.6%. Hypogammaglobulinemia was diagnosed in 43.8% of the patients, and it was significantly more prevalent among males and the 375 mg/m^2^ and 500 mg doses. Hematological malignancy was the only predictor for infection. Patients with blood type AB or B, hematological malignancies, and corticosteroids had a significantly higher mortality rate. Receiving the 1000 mg dose and having a low CD19 were associated with a significantly lower risk of infection and mortality, respectively. Conclusions: Hypogammaglobulinemia was diagnosed in 43.8% of the patients, and it was significantly more common among males and the 375 mg/m^2^ and 500 mg doses. Hematological malignancies were significantly associated with higher infection and mortality rates, while corticosteroids were significantly associated with a higher mortality. Since the culprit of mortality was infection, these findings highlight the critical need for more frequent immunological monitoring during rituximab treatment period to mitigate the burden of infection and identify candidates for immunoglobulin replacement.

## 1. Introduction

Monoclonal antibodies are highly specific immunotherapies designed to interact with a single molecule [1]. Rituximab (RTX) is a human monoclonal antibody directed against the B-cell transmembrane protein CD20 [2]. Once bound, RTX triggers antibody-dependent cell-mediated cytotoxicity, complement-dependent cytotoxicity, phagocytosis via the reticuloendothelial system, and apoptosis, depleting CD20-positive B-cells [3,4]. RTX has been widely used in the treatment of several conditions, including hematological malignancies such as non-Hodgkin’s lymphoma, connective tissue diseases such as rheumatoid arthritis, vasculitis, and pemphigus vulgaris, demyelinating diseases such as multiple sclerosis and neuromyelitis optica, glomerular diseases such as steroid-resistant nephrotic syndrome, recurrent focal segmental glomerulosclerosis, and membranous nephropathy, and primary immunodeficiency [4,5,6,7]. 

Given its route of administration, RTX commonly causes infusion-related reactions such as fever, chills, rigors, headache, dizziness, pruritus, urticaria, hypotension, and, rarely, serum sickness [3,8]. However, RTX is implicated in serious adverse effects as it depletes B-cells, resulting in a transient but prolonged state of severe immunosuppression, especially in patients with malignancies, and when given with other immunosuppressants such as corticosteroids [3,9]. Since RTX-induced B-cell depletion lasts up to 12 months, the risk of opportunistic infections such as pneumocystis jirovecii pneumonia, JC virus-induced progressive multifocal leukoencephalopathy, and reactivation of hepatitis B virus increases significantly [10,11,12].

It has been established that the risk of infection among RTX receivers might be related to immunoglobulin levels [13]. Hypogammaglobulinemia has been reported in 42–47.5% of patients treated with RTX [13,14]. Because of that, and to mitigate the aforementioned consequences of B-cell depletion, many experts, including the Rituximab Consensus Expert Committee, the British Society for Rheumatology, and the European League Against Rheumatism, recommend measuring the immunoglobulin levels before the initiation of and before each RTX cycle [15]. In addition, measuring immunoglobulin levels has therapeutic indications, as those with hypogammaglobulinemia who do not respond to vaccination might be candidates for immunoglobulin replacement [13,16]. 

Despite these recommendations, based on a study that included over 4000 patients, 85% of the patients treated with RTX did not have baseline immunoglobulin levels before its initiation [13]. This means there is little awareness about RTX’s effect on immunoglobulin levels and subsequent infection and mortality. Therefore, in this study, we retrospectively aimed to evaluate the rate and predictors of hypogammaglobulinemia and assess morbidity, in the form of severe infection requiring intensive care unit (ICU) admission, and mortality associated with RTX use in one of the largest medical centers in Saudi Arabia.

## 2. Method

### 2.1. Aim

To evaluate infection risk, mortality, and hypogammaglobulinemia and their associated factors among RTX receivers.

### 2.2. Study Setting and Design

This was a single-center retrospective cohort study conducted at King Abdulaziz Medical City (KAMC), Department of Medicine, Ministry of National Guard-Health Affairs, Riyadh, Kingdom of Saudi Arabia. KAMC is an academic government-funded tertiary hospital that combines clinical care, training, research academics, and state-of-the-art medical technologies.

### 2.3. Inclusion and Exclusion Criteria

All adult patients (aged > 18 years) who were treated with RTX at KAMC from January 2016 to December 2020 were included. Patients with an unclear diagnosis or those who met the definition of hypogammaglobinemia solely based on IgA were excluded.

### 2.4. Data Collection

The required data were obtained by screening the electronic medical records (via the KAMC electronic system “BestCare” Seoul, South Korea: ezCaretech Co) of all the patients who were treated with RTX for any reason since 2016. The following data were collected: age; sex; blood group; body mass index (BMI); comorbidities such as diabetes mellitus (DM), hypertension (HTN), dyslipidemia (DLP), chronic kidney disease (CKD), hematological malignancies, multiple sclerosis (MS), myasthenia gravis (MG), connective tissue disease (CTD), glomerular diseases, hypothyroidism, chronic pulmonary disease, ischemic heart disease or coronary artery disease, cardiac arrythmia, and stroke; history of transplant (solid organ or hematopoietic stem cell transplant); some lab values such as hemoglobin A1C and creatinine level; the diagnosis for which RTX was used; RTX dose (375 mg/m^2^, 500 mg, or 1000 mg) and frequency; other immunosuppressants or immunomodulators; immunoglobulin levels (IgG, IgM, and IgA); CD19 level; history of infection during RTX treatment period; need for ICU admission; outcomes (survival or death); and cause of death.

Hypogammaglobulinemia was defined as a cut-off value below the normal limit, depicted by an IgG level of <7.51 g/L, IgM level of <0.46 g/L, and/or IgA level of <0.82 g/L. Severe infection was defined as any infection that required ICU admission.

### 2.5. Statistical Analysis

The Statistical Package for the Social Sciences (SPSS version 28; IBM Co., Armonk, NY, USA) was used for data analysis. The categorical data are presented as frequency and percentage (%), the numerical parametric data as mean ± standard deviation (SD), and the numerical non-parametric data as median and interquartile range (IQR). The independent sample *t*-test was used to analyze the numerical variables, whereas the chi-square test or Fisher’s exact test, when appropriate, was used to analyze the categorical variables. Logistic regression was performed to assess the association between different factors and severe infection requiring ICU admission. Cox proportional hazards regression was performed to assess the effect of different factors on the risk of mortality, given that the patients had survived up to a specific time. A two-tailed *p* value < 0.05 was considered statistically significant.

### 2.6. Ethical Consideration

The study was approved by the Institutional Review Board of King Abdullah International Medical Research Center, Ministry of National Guard Health Affairs, Riyadh, Kingdom of Saudi Arabia (NRC21R/386/09). Informed consent was waived because of the retrospective nature of this study. Access to the data was restricted to the researchers. The confidentiality of all patients was protected, and no names or medical record numbers were used. Privacy and confidentiality were assured, and all the hard and soft copies of data were kept in a secure place within the Ministry of National Guard Health Affairs premises.

## 3. Results

A summary of patients’ baseline characteristics is shown in Table 1 and Table 2. There was a total of 137 patients on RTX. More than half (54%; *n* = 74) of the patients were female, with a mean age of 47.69 ± 18.86 years and an average BMI of 28.57 ± 6.55 kg/m^2^. The majority (56.9%; *n* = 78) of the patients were younger than 50 years old, and only around a third (16.1%; *n* = 22) were older than 70 years old. Almost two-quarters (41.6%; *n* = 57) of the patients were obese. The most common blood type was AB (36.5%; *n* = 50), followed by A (20.4%; *n* = 28), B (13.1%; *n* = 18), and O (4.4%; *n* = 6).

**Figure 1:**
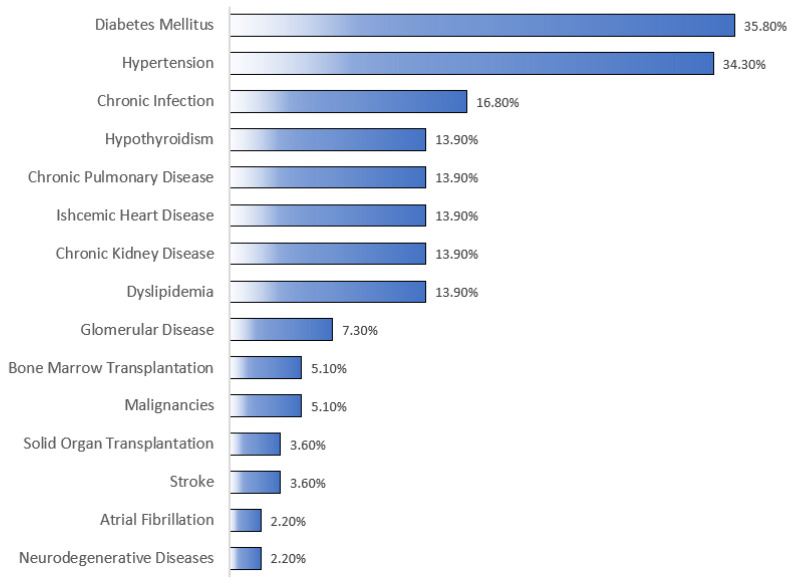
Comorbid conditions of the studied patients. As shown in the figure, the most common associated comorbidities were diabetes mellitus and hypertension, according for 35.8% and 34.3%, respectively. Malignancies refer to any malignancy for which RTX was not primarily used to treat.

As summarized in Table 2, the most frequent indications for which RTX was used were hematological malignancies, autoimmune CTD, and benign/non-malignant hematological diseases, accounting for 42.3%, 27%, and 17.5%, respectively. More than half (56.2%; *n* = 77) of the patients received the 375 mg/m^2^ dose, with a median number of five doses/cycles (Q1, Q3: 2, 7). RTX’s mean cumulative dose was 3216 ± 2282 mg, with a median of 2625 (Table 3). Besides RTX, almost half (48.9%; *n* = 67) of the patients were on steroids, and less than a third (10.2%; *n* = 14) were on methotrexate. As shown in Figure 1, the most notable associated comorbidities were DM, HTN, chronic infection, dyslipidemia, and CKD, accounting for 35.8%, 34.3%, 16.8%, 13.9%, and 13.9%, respectively. Only a few patients had a history of bone marrow (5.1%; *n* = 7) or solid organ (3.6%; *n* = 5) transplantation. The overall mortality rate was 22.6% (*n* = 31), with sepsis/septic shock (45.2%; *n* = 14) and COVID-19 infection (16.1%; *n* = 5) being the most common underlying causes of mortality (Table 4).

**Figure 2:**
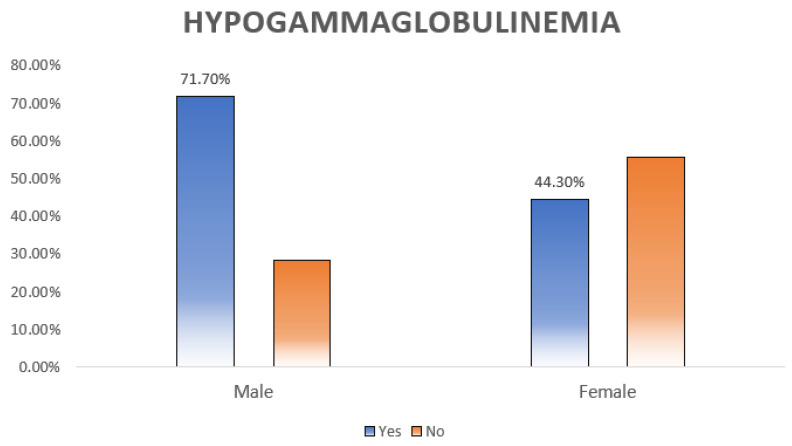
Relation between hypogammaglobulinemia and sex distribution. Almost three-quarters (71.1%) of the males had hypogammaglobulinemia compared to the females, and the difference was statistically significant (*p* = 0.005).

**Figure 3:**
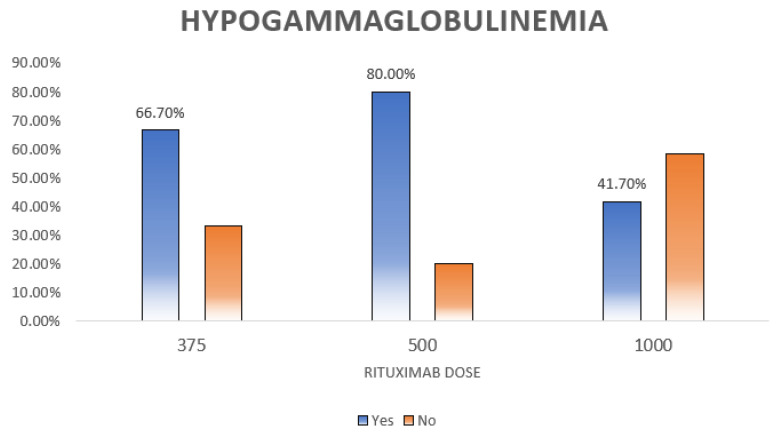
Relation between hypogammaglobulinemia and rituximab dose. This figure shows the distribution of hypogammaglobulinemia among different rituximab doses. The highest rate is seen for those who received the 500 dose and it is statistically significant (*p* = 0.012).

Hypogammaglobulinemia, defined as low IgG, IgM, or IgA, was diagnosed in 43.8% (*n* = 60) of the patients. As shown in Table 5 and Table 6, hypogammaglobulinemia was significantly more prevalent among males (*p* = 0.005) (Figure 2). Moreover, patients with different doses of RTX had different percentages of hypogammaglobulinemia (*p* = 0.012) (Figure 3).

In multivariable analysis, patients with hematological malignancies had significantly higher odds of developing a severe infection and being admitted to the ICU than those with no hematological malignancies (OR = 17.77, 95% CI: 1.29 to 245.62, *p* = 0.032). On the other hand, patients who received the 1000 mg dose of RTX had significantly lower odds of having severe infection and being admitted to the ICU (OR = 0.04, 95% CI: 0 to 0.8, *p* = 0.036) compared to those who received the 375 mg/m^2^ dose, as summarized in Table 7.

**Figure 4:**
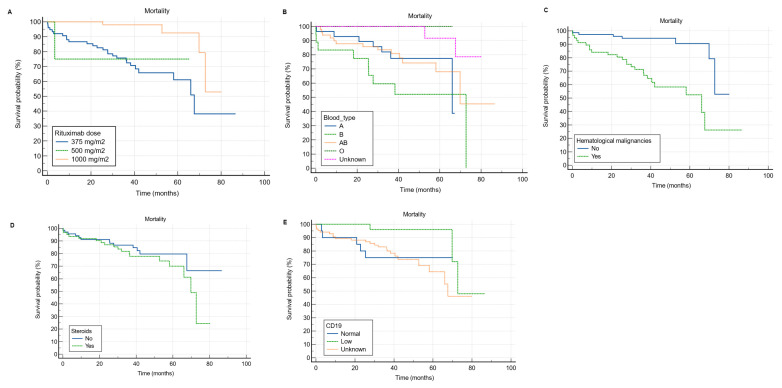
Kaplan–Meier curves for overall survival analysis according to rituximab dose, blood type, the presence of hematological malignancy, steroid use, and CD19. (**A**) receiving the 1000 mg RTX dose was significantly associated with lower risk of mortality compared to the 375 mg/m^2^. (**B**) it appears that patients with blood type B and AB have worse survival compared to blood type A. (**C**) hematological malignancies significantly hinder the survival of RTX receivers. (**D**) the concurrent use of steroid among RTX receivers significantly hinders the survival. (**E**) It appears that patients with low CD19 have better survival compared to those with normal levels.

As shown in Table 8, based on univariate Cox regression analysis, patients with hematological malignancies had a significantly higher risk of mortality than those with no hematological malignancies (HR = 6.18, 95% CI: 2.63 to 14.55, *p* < 0.001). However, patients who received RTX in the 1000 mg dose had a significantly lower risk of mortality (HR = 0.15, 95% CI: 0.05 to 0.44, *p* = 0.001) compared to those who received the 375 mg/m^2^ dose (Figure 4A). In multivariable Cox regression analysis, patients with blood type B (HR = 6.82, 95% CI: 2 to 23.22, *p* = 0.002) and AB (HR = 1.58, 95% CI: 0.5 to 4.98, *p* < 0.001) had a significantly higher risk of mortality compared to those with blood type A (Figure 4B). Additionally, patients with hematological malignancies had a significantly higher probability of mortality than those with no hematological malignancies (HR = 11.74, 95% CI: 2.18 to 63.14, *p* = 0.004) (Figure 4C). Moreover, patients on steroids had a significantly higher risk of mortality than those who were not on steroids (HR = 3.12, 95% CI: 1.24 to 7.83, *p* = 0.015) (Figure 4D). In comparison to patients with normal CD19, those with low CD19 had a significantly lower risk of mortality (HR = 0.08, 95% CI: 0.01 to 0.74, *p* = 0.026) (Figure 4E).

## 4. Discussion

B cells are derived from hematopoietic stem cells in the bone marrow [17]. Through a sophisticated mechanism, these cells recognize pathogens and differentiate into antibody-producing plasma cells [18]. RTX is a human monoclonal antibody targeted against the B-cell surface marker CD20 [2]. In this study, we evaluated infection risk, mortality rate and predictors, and hypogammaglobulinemia prevalence and associated factors among 137 patients treated with RTX for a variety of clinical indications in one of the largest medical centers in Saudi Arabia.

In this study, the patients’ mean age was 47.69 ± 18.86 years, which is younger than what has been reported in the literature [13,19]. This might be explained by the fact that our patients had heterogeneous diagnoses. To clarify, the most common indication for RTX use were hematological malignancies, followed by autoimmune CTD, non-malignant hematological conditions, glomerular diseases, and neurological conditions such as MS and MG. However, most of the available literature addressing RTX and the risk of infection and mortality include a specific disease such as rheumatoid arthritis, or a group of diseases such as autoimmune diseases [14,19,20,21]. This may also explain the equal sex distribution in our study.

In the current study, infection was observed in over a third (32.8%; *n* = 45) of the patients, but only a third (33%; *n* = 14) of those who were infected had a severe infection necessitating ICU admission. It is well known that immunosuppressant-induced severe infection is associated with old age, kidney impairment, chronic cardiopulmonary diseases, organ or hematopoietic stem cell recipients, and DM [22,23,24,25]. In the present study, DM (35.8%; *n* = 49) and HTN (34.3%; *n* = 47) were the most frequent comorbid conditions, with only a few having a chronic infection, mainly hepatitis B or C, dyslipidemia, CKD, or chronic cardiopulmonary diseases. Additionally, a small proportion of the patients had bone marrow (5.1%; *n* = 7) or solid organ transplants (3.6%; *n* = 5). Regardless, it is hard to compare our results with the literature owing to the difference in defining a severe infection. Several studies defined an infection as severe once it required hospitalization or IV antibiotics [6,13,14,19,20,21,22]. In our study, however, it was defined as any infection requiring ICU admission, which happened in only 10.21% (*n* = 14) of the patients. Based on a study that evaluated infection risk in 4479 patients treated with RTX for various indications, almost a third (28.2%) had severe infections, mainly in the first 6 months of RTX initiation [13]. A trial that evaluated the long-term safety and efficacy of RTX in combination with belimumab in 15 patients with systemic lupus erythematosus reported three major infections (20%), requiring hospitalization, and eight minor infections (53.3%) [26]. Another study evaluated the risk of infection among 1681 rheumatoid arthritis patients treated with RTX and reported that only 5% developed a severe infection requiring hospitalization, IV antibiotics, or resulting in death [19]. Additionally, a study that assessed infection rates among 147 patients with ANCA-positive vasculitis treated with RTX found a total of 88 (59.9%) infection events, almost a third (29.5%; *n* = 26) of which were identified as severe requiring IV antibiotics or hospitalization [22]. The discrepancy in the definition of a severe infection, the different indications for RTX use, and the various immunosuppressants given with RTX make it challenging to determine the infection risk among RTX receivers.

Since its inception, RTX has reconstituted the treatment and redirected the survival of B-cell malignancies, including diffuse large B-cell lymphoma, follicular lymphoma, and mantle cell lymphoma [27]. In the present study, the most common primary diagnosis for which RTX was used was hematological malignancy, and it was significantly associated with higher infection (*p* = 0.032) and mortality (*p* = 0.004) rates. We believe that these worrisome findings are probably attributed to the fact that RTX is usually given with other immunosuppressants such as methotrexate, azathioprine, cyclophosphamide, and mycophenolate or as a part of treatment regimens such as R-CHOP, which includes cyclophosphamide, doxorubicin, vincristine, and prednisone, and R-EPOCH, which includes etoposide, prednisone, vincristine, cyclophosphamide, doxorubicin, in addition to RTX. Additionally, around half (48.9%; *n* = 67) of our patients were on corticosteroids, and their use was significantly associated with a higher mortality rate (*p* = 0.015). The studied population has an impaired humoral immunity due to RTX use, and adding another immunosuppressant such as azathioprine, mycophenolate, or cyclophosphamide would impair cell-mediated immunity [28]. Furthermore, steroids are powerful anti-inflammatory agents that disrupt innate immunity as well [29]. Impairment of both innate and adaptive immunity makes these patients vulnerable to severe and fulminant infections and increases their mortality.

We also found a statistically significant association between the 375 mg/m^2^ RTX dose and both infection and mortality. The former can be explained with the same aforementioned explanation, as the 375 mg/m^2^ dose weekly for 4 weeks or every 3–4 weeks is the one used in lymphoma treatment. Moreover, in addition to therapy-related factors, the pathogenesis of the primary disease plays a crucial role in susceptibility to infection and subsequent mortality in these patients [30]. Also, we found that patients who received the 1000 mg RTX dose had a significantly lower risk of mortality compared to those who received the 375 mg/m^2^ dose. The reason might be related to the primary diagnosis for which RTX was used, frequency of RTX administration, and the concurrent use of other immunosuppressants. To clarify, the 1000 mg dose is commonly used in the treatment of neurological diseases such as MG and MS. Those patients do not typically require multiple immunosuppressants and are usually young and relatively healthy with no or only mild comorbidities compared to those with hematological malignancies. Additionally, most of those patients were not on steroids as they are often only used for acute relapses and crises. This could be another possible explanation for the observed favorable outcomes in patients receiving the 1000 mg dose compared to the 375 mg/m^2^ dose.

Our results revealed a mortality rate of 22.6% (*n* = 31), with infection (61.3%; *n* = 19) being the most frequent cause of mortality, consistent with the literature [31]. Since its emergence, an important cause of mortality, especially in immunocompromised individuals such as the studied population, has been COVID-19 infection, which was the underlying cause of mortality in 16.1% (*n* = 5) of our patients [32,33]. Compared to the general population, B-cell-depleted individuals need a longer time to clear the virus, and because of that, COVID-19 infection is more likely to persist and last for months [34]. Furthermore, total hospitalization time and COVID-19 complications, including respiratory failure and ICU admission, occur at higher rates among these patients [34,35,36]. Another dilemma is the fact that RTX affects memory B cells in addition to effector B cells, possibly blunting the response to the COVID-19 vaccine [34]. Since the most frequently observed indications for RTX use in our study were hematological malignancies (42.3%; *n* = 58) and autoimmune CTDs (27%; *n* = 37), we will discuss the effect of COVID-19 infection on the outcomes of these two groups of diseases. To begin with, based on a systematic review and meta-analysis that investigated COVID-19 morbidity and mortality in cancer patients, the risk of severe COVID-19 infection and mortality increases by 2.84- and 2.60-fold, respectively, among cancer patients [37]. More specifically, several studies have also found that hematological malignancies compromise the outcomes of COVID-19 infection and the response to the COVID-19 vaccine, and receiving RTX within a year of the vaccine significantly reduces antibody production and, therefore, attenuates the efficacy of the vaccine [38,39]. Unfortunately, this is not limited to the COVID-19 vaccine; it has also been observed with the pneumococcal and influenza vaccines [16]. Similarly, a study that evaluated the outcomes of COVID-19 in 122 patients with a heterogeneous group of inflammatory CTDs found that RTX use was associated with significantly longer hospitalization and higher mortality [35].

Hypogammaglobulinemia is defined as low serum immunoglobulin levels [40]. Although it can be primary, hypogammaglobulinemia is frequently diagnosed secondary to medical conditions, such as nephrotic syndrome and infections, and medications, such as corticosteroids and immunomodulators like RTX used for various hematological malignancies, autoimmune CTDs, glomerular conditions, and neurological diseases [41,42]. In the present study, hypogammaglobulinemia was defined as a deficiency in either IgG, IgM, and/or IgA, and it was observed in 43.8% (with 95% CI: from 33.4 to 56.4) of our patients. This is in accordance with the literature, as several studies have reported a hypogammaglobulinemia rate ranging from 42 to 47.5% among RTX receivers [13,14,43,44,45,46]. This dose range not apply to all the studies, though; there are published studies with lower [6,47,48] or higher [21,49] percentages, but most of the literature reported a percentage within this range. Generally, patients with malignancies are more prone to developing hypogammaglobulinemia during or after treatment, compared to patients with non-malignant conditions. This might be accredited to the nature of cancer itself, as it is already a well-known cause of hypogammaglobulinemia, and to the aggressive treatment regimens used in cancer. To clarify, patients with B-cell lymphoma, for example, might need more than one course of RTX, and this has been linked to a higher risk of hypogammaglobulinemia [50]. We found a statistically significant relation between the development of hypogammaglobulinemia and the male sex (71.7%) as compared to females (44.3%) (*p* = 0.005). We believe that sex may not be directly related to hypogammaglobulinemia. Rather, male predominance observed in most hematological malignancies and the aggressive nature of autoimmune CTDs in males requiring RTX use might be the underlying cause for this finding [51,52,53]. Based on a large study of 4479 patients treated with RTX for various clinical conditions, the male sex was significantly associated with higher mortality particularly in hematological malignancy and CTD groups, supporting the previously mentioned explanation [13]. Likewise, a study that examined hypogammaglobulinemia and infection risk among 29 granulomatosis with polyangiitis patients treated with RTX found an association between the male sex and hypogammaglobulinemia [43].

We found that different doses of RTX had significantly (*p* = 0.012) different rates of hypogammaglobulinemia (Table 4); however, we could not find an association between cumulative RTX dose and hypogammaglobulinemia (*p* = 0.307). Comparably, a study of 243 patients treated with RTX for several multi-system autoimmune diseases find no association between cumulative RTX dose and hypogammaglobulinemia [21]. Opposite to our findings, a study of 103 patients with complicated nephrotic syndrome who received at least a single dose of RTX found an association between repeated RTX cycles and hypogammaglobulinemia [49]. Next, a study of 169 patients with neuromyelitis optica who were treated with RTX reported that the mean annual RTX dose was significantly associated with hypogammaglobulinemia [44]. We believe that this discrepancy in the results is largely attributed to several factors, including the primary disease and patients’ characteristics. It is also important to mention that some of the studies have significantly linked RTX dose and infection, but not RTX dose and hypogammaglobulinemia [14,20,44,50]. We could not establish an association between the number of RTX doses and hypogammaglobulinemia (*p* = 0.153). Additionally, we failed to prove an association between other immunosuppressants, such as methotrexate, azathioprine, and mycophenolate, or comorbidities and hypogammaglobulinemia. Some studies have linked some immunosuppressants, such as cyclophosphamide, corticosteroids, and mitoxantrone, with the development of hypogammaglobulinemia [16,50]. It is also worth mentioning that many studies have found a significant association between particular comorbidities, such as chronic pulmonary disease, heart failure, DM, and cancer, and severe infection, but not hypogammaglobulinemia, suggesting a highly complicated interplay of multiple factors related to the patients, their pathologies, and their treatment regimens [14,20,44,50].

In this study, the majority (86.13%; *n* = 118) of patients underwent B cell immunophenotyping, and over a quarter (24.57%; *n* = 29) of them had low CD19. Like immunoglobulin levels, which were not measured in over a quarter of our patients, CD19 was unchecked in almost a third (16.9%; *n* = 20) of the patients. Although the percentage of patients who did not undergo immunophenotyping was relatively low, we believe that the status quo can be improved. We also found that low CD19 was significantly associated with a lower risk of mortality (HR = 0.08, 95% CI: 0.01 to 0.74, *p* = 0.026). As expected, patients who receive appropriate doses of RTX should have low CD19, which is a surrogate for CD20. And, at least in theory, patients with incomplete B cell depletion, measured by CD19 level, should receive an extra dose of RTX [54]. An observational study investigating the efficacy of RTX in 71 patients with systemic lupus erythematosus found that patients with B cells in renal biopsy had poor outcomes [55]. Another study assessed the role of CD19 level in 42 patients with nephrotic syndrome and found a positive correlation between CD19 B cell percentage and risk of relapse [56]. Also, a study of 44 patients treated with RTX-based desensitization for ABO-incompatible kidney transplantation found that high CD19 significantly increased the risk of acute antibody-mediated rejection [57]. Although not exactly similar to our findings, we believe these findings explain the same concept, as low CD19 indicates that the patient received an appropriate dose of RTX, which controlled the primary disease and might explain the low mortality.

Unfortunately, despite the current recommendations, more than a quarter (21.9%; *n* = 30) of our patients did not have their immunoglobulin levels checked, and more than a third (16.9%; *n* = 20) of our patients did not have their CD19 level checked, reflecting a lack of awareness about the importance of immunological monitoring during the treatment period. We advocate for immunoglobulin monitoring and B cell immunophenotyping throughout the treatment period, especially in male patients with hematological malignancies and those using corticosteroids, to detect hypogammaglobulinemia early, identify immunoglobulin replacement candidates, and possibly mitigate the risk of infection and mortality.

The current study has several limitations. First, a sample size of 137 is considered small, especially among patients with heterogeneous diagnoses and different dosing regimens, which might be another limitation. Second, the retrospective nature of the study makes it challenging to establish a causative relation between the use of RTX and infection and/or mortality as both can be influenced by the underlying primary disease, comorbidities, and the concurrent use of other immunosuppressants. Moreover, we did not gather data about the time of infection following RTX initiation. It would be beneficial to know the median time of infection after RTX initiation. Next, hypogammaglobulinemia was not classified as mild, moderate, or severe. Instead, we collected immunoglobulin levels (IgG, IgM, and IgA) as a numerical variable and then coded the levels as either normal or low. It might have been more beneficial to know the fraction of patients with severe hypogammaglobulinemia and the associated factors. Also, we did not look at immunoglobulin levels before RTX initiation. This may have given us an idea of the awareness among different specialties about the importance of measuring immunoglobulin levels before RTX initiation. In addition, our definition of severe infection, which was any infection requiring ICU admission, might have underestimated the real percentage of infection among the studied population. Finally, some of the findings might not be clinically relevant or could not be well explained by the authors or the literature, such as the statistical significance between specific blood groups (B and AB) and mortality.

## 5. Conclusions

Hypogammaglobulinemia was diagnosed in 43.8% of the patients, and it was significantly more pronounced among males and the 375 mg/m^2^ and 500 mg RTX doses. Hematological malignancies were the only predictor of infection in this study. However, in addition to hematological malignancies, corticosteroid use and blood types B and AB were associated with higher odds of mortality among RTX receivers. Since the predominant underlying cause of mortality was infection in almost three-thirds of the patients, these findings emphasize the significance of more frequent immunological monitoring throughout the treatment period to possibly prevent or mitigate the consequences of infection and identify candidates for immunoglobulin replacement.

## Figures and Tables

**Table 1:** Baseline characteristics of the studied patients (*n* = 137).

	N	%
Age (years)		
≤30 years	31	22.6
31–50 years	47	34.3
51–70 years	37	27.0
≥71 years	22	16.1
Gender		
Male	63	46.0
Female	74	54.0
BMI		
Underweight (<18.5)	3	2.2
Normal (18.5–<25)	40	29.2
Overweight (25.0–<30)	37	27.0
Obese (>30)	57	41.6
Blood Type		
A	28	20.4
B	18	13.1
AB	50	36.5
O	6	4.4
Unknown	35	25.5
Rhesus (Rh) Factor		
Positive	9	6.6
Negative	93	67.9
Unknown	35	25.5

BMI: Body mass index.

**Table 2:** Primary diagnosis and medication use of the studied patients (*n* = 137).

	N	%
The primary diagnosis for which rituximab was used		
Hematological malignancies	58	42.3
Autoimmune connective tissue disease	37	27.0
Benign/non-malignant hematological disease	24	17.5
Glomerular disease (nephrotic/nephritic syndrome)	10	7.3
Demyelination disorder (mainly MS)	9	6.6
Autoimmune inflammatory disease of the nervous system (mainly MG)	6	4.4
Rituximab dose		
375 mg/m^2^	77	56.2
500 mg	6	4.4
1000 mg	54	39.4
Number of rituximab doses		
Median (IQR)	5 (3–7)	
Min–Max	1–36	
Other immunosuppressants		
Steroids	67	48.9
Methotrexate	14	10.2
Azathioprine	13	9.5
Mycophenolate	12	8.8
Hydroxychloroquine	10	7.3
Other medications	57	41.6

IQR: Interquartile range.

**Table 3:** The cumulative dose of different dosing regimens for RTX.

		Hypogammaglobulinemia		*p*-Value
		No	Yes	
Cumulative Dose	The whole sample	3497 (2426)	3069 (1896)	0.307
	375 mg/m^2^	2021 (1456)	2615 (1612)	0.194
	500 mg *			
	1000 mg	4500 (2472)	4100 (2100)	0.560

* Cannot be compared as this group had only one case with hypogammaglobulinemia.

**Table 4:** Outcomes of the studied patients (*n* = 137).

	N	%
Outcome		
Alive	97	70.8
Dead	31	22.6
Unknown	9	6.6
Cause of Death (*n* = 31)		
Sepsis/septic shock	14	45.2
COVID-19	5	16.1
Progression of primary disease	7	22.6
Unknown/unclear	5	16.1

**Table 5:** Association between hypogammaglobulinemia and different risk factors.

	Non-Hypogammaglobulinemia	Hypogammaglobulinemia	*p* Value
Age (years)	45.81 ± 19.83	49.57 ± 18.2	0.311
BMI (kg/m^2^)	27.48 ± 6.08	27.23 ± 6.03	0.833
Gender			
Male	13 (28.3%)	33 (71.7%)	**0.005**
Female	34 (55.7%)	27 (44.3%)	
Blood Type			
A	10 (50.0%)	10 (50.0%)	0.825
B	6 (37.5%)	10 (62.5%)	
AB	14 (37.8%)	22 (62.2%)	
O	2 (50.0%)	2 (50.0%)	
HgbA1C (%)	6.38 ± 1.36	6.81 ± 1.48	0.258
BMT	1 (2.1%)	5 (8.3%)	0.227
Rituximab dose			
375 mg/m^2^	18 (33.3%)	37 (66.7%)	**0.012**
500 mg	1 (20.0%)	4 (80.0%)	
1000 mg	28 (58.3%)	19 (41.7%)	
Number of rituximab doses	4.83 ± 3.06	5.82 ± 3.83	0.153
Other Immunosuppressants			
Steroids			
Yes	24 (45.3%)	29 (54.7%)	0.779
No	23 (42.6%)	31 (57.4%)	
Methotrexate			
Yes	2 (22.2%)	7 (77.8%)	0.293
No	45 (45.9%)	53 (54.1%)	
Azathioprine			
Yes	7 (58.3%)	5 (41.7%)	0.286
No	40 (42.1%)	55 (57.9%)	
Mycophenolate			
Yes	4 (33.3%)	8 (66.7%)	0.433
No	43 (45.3%)	52 (54.7%)	
Hydroxychloroquine			
Yes	5 (50.0%)	5 (50.0%)	0.502
No	42 (43.3%)	55 (56.7%)	
Other medications			
Yes	16 (37.9%)	27 (62.8%)	0.251
No	31 (48.4%)	33 (51.6%)	

BMI: Body mass index, HgbA1C: Hemoglobin A1C, BMT: Bone marrow transplant. Bold: statistical significance at *p* value < 0.05.

**Table 6:** Comparison of severe infection and mortality based on the presence of hypogammaglobulinemia.

	Non-Hypogammaglobulinemia	Hypogammaglobulinemia	*p* Value
Severe Infection			
No	41 (87.2%)	52 (86.7%)	0.931
Yes	6 (12.8%)	8 (13.3%)	
Outcome			
Alive	34 (72.3%)	40 (66.7%)	0.272
Dead	8 (17.0%)	17 (28.3%)	
Unknown	5 (10.6%)	3 (5.0%)	

No statistically significant difference was observed between the two groups regarding mortality or severe infection.

**Table 7:** Logistic regression model for factors associated with severe infection requiring ICU admission.

	Univariate			Multivariable		
	uOR	95% CI	*p* Value	aOR	95% CI	*p* Value
Autoimmune CTD	2.23	0.72 to 6.92	0.167	5.54	0.33 to 94.32	0.237
Hematological malignancies	1.95	0.64 to 5.95	0.243	17.77	1.29 to 245.62	**0.032**
Benign/non-malignant hematological disease	1.32	0.34 to 5.16	0.685	0.91	0.11 to 7.65	0.928
CNS autoimmune disease	0.6	0.07 to 4.93	0.634	54.9	0.66 to 4543.02	0.075
Glomerular disease	0.97	0.11 to 8.32	0.981	3.3	0.04 to 243.09	0.586
Rituximab dose						
375 mg/m^2^	Ref					
500 mg	3.35	0.54 to 20.73	0.194	1.91	0.09 to 42.04	0.682
1000 mg	0.26	0.05 to 1.23	0.089	0.04	0 to 0.8	**0.036**
Number of rituximab doses	1	0.88 to 1.13	0.964	0.85	0.67 to 1.09	0.199
Hypogammaglobulinemia						
No	Ref					
Yes	1.05	0.34 to 3.27	0.931	0.49	0.1 to 2.44	0.386
Unknown	---	---	---	---	---	---
CD19						
Normal	Ref					
Low	0.65	0.12 to 3.63	0.627	1.79	0.16 to 20.32	0.64
Unknown	0.57	0.14 to 2.36	0.435	0.56	0.064 to 4.93	0.602
Steroids	2.89	0.86 to 9.73	0.086	2.61	0.49 to 13.72	0.258
Methotrexate	1.54	0.31 to 7.72	0.598	4.73	0.49 to 45.81	0.18
Azathioprine	3.08	0.74 to 12.89	0.123	4.51	0.43 to 47.15	0.208
Mycophenolate	3.45	0.81 to 14.66	0.093	5.86	0.43 to 79.78	0.185
Hydroxychloroquine	0.97	0.11 to 8.32	0.981	0.74	0.01 to 107.24	0.907

uOR: Unadjusted odds ratio, aOR: Adjusted odds ratio, CI: Confidence interval. Bold: statistical significance at *p* value < 0.05. ---: No estimates are reported due to shortage of events leading to imprecise estimates with confidence interval ranging to infinity.

**Table 8:** Univariate and multivariable Cox proportional hazards regression for overall survival analysis.

	Univariate			Multivariable		
	HR	95% CI	*p* Value	HR	95% CI	*p* Value
Blood Type						
A	Ref					
B	2.19	0.8 to 5.99	0.127	6.82	2 to 23.22	**0.002**
AB	0.93	0.37 to 2.36	0.881	1.58	0.5 to 4.98	**<0.001**
O	---	---	---	---	---	---
Unknown	0.16	0.03 to 0.81	**0.027**	0.36	0.05 to 2.48	0.299
Autoimmune CTD	0.41	0.15 to 1.09	0.075	0.88	0.15 to 5.14	0.887
Hematological malignancies	6.18	2.63 to 14.55	**<0.001**	11.74	2.18 to 63.14	**0.004**
Benign/non-malignant hematological disease	1.49	0.61 to 3.65	0.384	1.24	0.35 to 4.39	0.739
CNS autoimmune disease	0.21	0.03 to 1.54	0.124	1.12	0.08 to 14.74	0.934
Glomerular disease	0.55	0.07 to 4.05	0.556	2.97	0.22 to 39.63	0.411
Rituximab dose						
375 mg/m^2^	Ref					
500 mg	0.95	0.13 to 7.05	0.961	1.03	0.03 to 32.75	0.985
1000 mg	0.15	0.05 to 0.44	**0.001**	0.66	0.1 to 4.18	0.66
Number of rituximab doses	1.06	0.99 to 1.13	0.086	0.97	0.86 to 1.09	0.563
Antibodies						
Non-hypogammaglobulinemia						
Hypogammaglobulinemia	1.36	0.58 to 3.16	0.478	0.7	0.25 to 2.01	0.512
Unknown	1.27	0.44 to 3.71	0.66	0.57	0.15 to 2.2	0.412
CD19						
Normal	Ref					
Low	0.31	0.07 to 1.34	0.116	0.08	0.01 to 0.74	**0.026**
Unknown	1.16	0.44 to 3.08	0.763	0.29	0.07 to 1.28	0.102
Steroids	1.51	0.74 to 3.08	0.261	3.12	1.24 to 7.83	**0.015**
Methotrexate	0.65	0.15 to 2.74	0.558	−0.82	0.08 to 2.4	0.344
Azathioprine	0.74	0.18 to 3.12	0.681	−0.28	0.09 to 6.6	0.803
Mycophenolate	0.64	0.15 to 2.73	0.544	−0.52	0.08 to 4.43	0.614
Hydroxychloroquine	0.26	0.03 to 1.95	0.189	0.75	0.15 to 30.18	0.579

CI: Confidence interval. Bold: statistical significance at *p* value < 0.05. ---: No estimates are reported due to shortage of events leading to imprecise estimates with confidence interval ranging to infinity.

## Data Availability

The data presented in this study is available on request from the corresponding author.

## Abbreviations

RTX: Rituximab, CD20: Cluster of differentiation 20, ICU: Intensive care unit, KAMC: King Abdulaziz Medical City, BMI: Body mass index, DM: Diabetes mellitus, HTN: Hypertension, DLP: Dyslipidemia, CKD: Chronic kidney disease, MS: Multiple sclerosis, MG: Myasthenia gravis, CTD: Connective tissue disease.
